# Multicenter prospective observational study on hospital pharmacist interventions to reduce inappropriate medications

**DOI:** 10.3389/fphar.2023.1195732

**Published:** 2023-06-29

**Authors:** Shinya Suzuki, Mayako Uchida, Hideki Sugawara, Yukio Suga, Takayuki Nakagawa, Hisamitsu Takase

**Affiliations:** ^1^ Department of Pharmacy, National Cancer Center Hospital East, Kashiwa, Japan; ^2^ Research Promotion Committee, Japanese Society for Pharmaceutical Palliative Care and Sciences (JSPPCS), Osaka, Japan; ^3^ Department of Education and Research Center for Pharmacy Practice, Faculty of Pharmaceutical Sciences, Doshisha Women’s College of Liberal Arts, Kyoto, Japan; ^4^ Department of Pharmacy, Kagoshima University Hospital, Kagoshima, Japan; ^5^ Department of Clinical Drug Informatics, Faculty of Pharmacy, Institute of Medical, Pharmaceutical and Health Science, Kanazawa University, Kanazawa, Japan; ^6^ Department of Clinical Pharmacology and Therapeutics, Kyoto University Hospital, Kyoto, Japan; ^7^ Department of Clinical Pharmacology and Pharmacotherapy, Faculty of Pharmaceutical Sciences, Wakayama Medical University, Wakayama, Japan; ^8^ Department of Pharmacy, Nippon Medical School Tama-Nagayama Hospital, Tokyo, Japan

**Keywords:** hospital pharmacists, intervention, inappropriate medications, polypharmacy, multicenter prospective observational study

## Abstract

**Background:** In Japan, the involvement of hospital pharmacists in inappropriate medications (IMs) practices has not been sufficiently reported. Therefore, this prospective study described the interventions of hospital pharmacists in discontinuing inappropriate drugs or reducing drug doses.

**Methods:** We conducted a prospective, multicenter, observational study to investigate the intervention of hospital pharmacists in inappropriate prescriptions for inpatients in September 2018. Fifty pharmacists from 45 hospitals in Japan participated in this study. IMs were defined as medications that pharmacists deemed inappropriate for patient treatment. The subjects of the study were patients who interacted with the participating pharmacists.

**Results:** During the study period, the median number of beds in hospitals where the 50 participating pharmacists worked was 380, and the average number of beds for which the pharmacists were responsible was 49. The enrolled hospital pharmacists recommended that doctors discontinue or reduce the doses of their regular drugs for 347 out of 1,415 (24.5%) patients. Among the 391 pharmacists’ recommendations to reduce IMs for 347 patients, physicians accepted 368 (94.1%) recommendations, and 523 drugs were discontinued as a result. Pharmacist intervention also led to improvements in hypnotic sedation, delirium, and hypotension. The most common reasons for IMs identified by pharmacists were “long-term administration of irresponsible or aimless medications” (44.5%), “adverse effects caused by medications” (31.5%), and “medications-mediated duplication of the pharmacological effect” (15.3%). Approximately 90% of pharmacists’ suggestions to reduce medications were accepted for each reason. The average number of regular medications used by patients involved in drug reduction was 8.2, and the average number of medications reduced was 1.7. A sub-analysis showed that patients using opioids tended to take more medications, and these patients were able to reduce the amount of medications taken. Interventions by pharmacists certified in palliative pharmacies tended to reduce adverse drug events.

**Conclusion:** This was the first multicenter prospective observational study conducted in Japan to demonstrate hospital pharmacist intervention’s effectiveness in promoting appropriate prescription and, consequently, a reduction in the number of medications in use and polypharmacy.

## 1 Introduction

Prescription errors are clinically meaningful errors that occur when prescribing decisions or formulary processes, resulting in an unintentional and significant decrease in the probability of timely and effective treatment or an increased risk of harm compared to generally accepted practice (Abdel-Qader et al., 2010) and can occur in daily clinical practice (Bates et al., 1995; Noguchi et al., 2016). Therefore, preventing prescription errors is essential for hospitals, and hospital pharmacists play a vital role in comprehensively intervening in patient care to prevent adverse drug events (ADEs) (Nester and Hale, 2002; Bond and Raehl, 2007; Abu-Naser, 2021).

The number of medications prescribed to patients increases with age and comorbidities (Park et al., 2016). Polypharmacy, defined as the inappropriate use of multiple medicines in patients in the 1960s (Canadian Medical Association, 1966), is associated with various problems, such as drug interactions, ADEs, increased medical expenses, and decreased medication adherence (Hersh et al., 2017). Studies have found that there is a dose-dependent relationship between polypharmacy and mortality, and excessive polypharmacy, such as regular use of ten or more medications, can lead to death (Leelakanok et al., 2017). However, increasing the number of drugs essential for a patient’s health can result in polypharmacy. Several studies have defined polypharmacy as the use of more drugs than is clinically indicated (Fulton and Allen, 2005). Recent research suggests that deprescribing, a process of identifying and discontinuing inappropriate medications (IMs), can reduce inappropriate polypharmacy in older patients (Reeve et al., 2015). However, it remains unclear whether deprescribing can improve clinical outcomes (Reeve et al., 2014; Scott et al., 2015). The use of multiple drugs in various healthcare settings is associated with potentially inappropriate medication (Nothelle et al., 2017; Nothelle et al., 2019).

According to Japan’s guidelines for the appropriate use of drugs for older patients, polypharmacy is a condition in which various problems occur due to the use of multiple drugs (Ooi, 2019). In Japan, there have been reports on the management of polypharmacy in a specific area of a single hospital (Hashimoto and Tensho, 2016; Horii and Atsuda, 2020). However, it resulted from medical staff with high-level knowledge, and it was difficult to generalize the data. As such, there is insufficient evidence of pharmacists’ contributions to addressing this problem in daily practice. To fill this gap, the Japanese Society for Pharmaceutical Palliative Care and Sciences (JSPPCS) Research Committee previously conducted a questionnaire survey on polypharmacy for members who worked as hospital pharmacists (Uchida et al., 2019) and community pharmacists (Suzuki et al., 2019). These results show the prevalence of polypharmacy and the benefits of pharmacy interventions for drug-related problems (Suzuki et al., 2019; Uchida et al., 2019).

However, since the findings were obtained from a retrospective observational study, the data lacked information on the patient background (age, disease, etc.), the actual number of cases of pharmacist recommendations and acceptance, detailed information such as discontinuation of IMs or reduction of drug doses following pharmacist recommendations, and information linking pharmacist interventions to each patient to improve the symptoms of ADEs. Furthermore, to date, no multicenter prospective studies have been conducted on deprescribing interventions by pharmacists. Therefore, to bridge this gap, the JSPPCS conducted a multicenter, prospective study to clarify the benefits of pharmacy interventions in reducing IMs in Japan. In our previous study, the questionnaire included analyses based on drug use status and professional certification. In this prospective study, we utilized the data obtained to perform a subanalysis, examining the impact of pharmacist intervention based on these factors (Suzuki et al., 2019; Uchida et al., 2019).

## 2 Materials and methods

### 2.1 Study design

A multi-center, prospective, observational study of pharmacists’ interventions on inappropriate prescriptions in a hospital was conducted in September 2018.

### 2.2 Participants

Hospital pharmacists who were members of the JSPPCS were recruited from the JSPPCS homepage to cooperate in this study. The recruitment method involved publicizing the prospective observational study on the JSPPCS website and inviting pharmacists who were interested in participating. A total of 50 pharmacists from 45 hospitals in Japan volunteered to take part in the study. Pharmacists who participated in the study reported information on patients who had pharmacy interventions that resulted in a reduction in the medications the patients were using.

### 2.3 Variables and data source

We conducted an analysis of various aspects, including the characteristics of the participating pharmacists and pharmacies, the nature of deprescribing through pharmacist interventions, the reasons behind and acceptance rates of pharmacists’ recommendations to reduce medications, the average number of drugs discontinued or reduced in dose, functional classification of discontinued drugs, and their pharmacological categories. The count of drugs discontinued or reduced in dose represents the total “number of cases” in which each drug was either discontinued or reduced, respectively.

At the outset of the survey, the participating pharmacists provided the following personal information: gender, years of experience as a pharmacist, working hours, duration dedicated to patient medication counseling, time spent on clinical pharmacy-related education, and details about their pharmacy, including the daily prescription count, total number of pharmacists employed, number of full-time staff pharmacists, percentage of patients with cancer, and number of patients availing pharmacy services. The pharmacist participants were provided with an electronic survey record form developed and validated by a research committee member of the JSPPCS (Supplementary Tables S1–S3). As part of their regular duties, pharmacists recorded their interventions during each patient visit. In addition, hospital pharmacists were required to record their work status and reduce the number of medicines used or doses administered during the study period.

During their intervention, pharmacists recorded the patient’s age and disease status. In cases in which a patient had more than one disease, the pharmacist recorded the comorbidities. In addition, the pharmacists recorded the following information on the survey record form daily for each case: the number of regularly used drugs in patients treated with inappropriate drugs, the timing when the pharmacist was aware of inappropriate prescriptions, the reasons for pharmacists’ recommendations to reduce medications, and the content of deprescribing, including the number and name of drugs discontinued and dose-reduced drugs by doctors. The study did not provide participating pharmacists with specific criteria for assessing ADEs, but they were instructed to use the CTCAEv4.0 as the criterion for assessing ADEs in their case report forms (CRFs). CTCAE stands for Common Terminology Criteria for Adverse Events; these criteria are also called “common toxicity criteria.” In CTCAE, an adverse event is defined as any abnormal clinical finding temporally associated with the use of a therapy. Specifically, pharmacists assessed whether ADEs were improved after deprescribing.

Subsequently, the JSPPCS office collected the records for analysis. We only collected data from the pharmacists who participated in the study. Even if they worked at the same hospital, those who did not participate in the study were excluded. After the observational period, we collected the data recorded by the enrolled pharmacists. The protocol was approved by the Institutional Review Board of the Ethics Committee of Osaka University of Pharmaceutical Sciences (approval no. 0052), and since the study used only administrative data to improve regular clinical practice, patient consent was waived. Consequently, while the patient data were anonymized for analysis, the pharmacists who conducted the pharmacy interventions were not anonymized.

### 2.4 Settings

This prospective, observational study focused on the 50 participating and the scope of practice with their respective institutions, where they implemented the pharmacy intervention.

### 2.5 Definition of inappropriate medications (IMs)

In this study, we categorized IMs as long-term administration of irresponsible or aimless medications, ADEs caused by medications, medication-mediated duplication of the pharmacological effect, medication-induced drug–drug interactions, inappropriate drugs for older patients, and inappropriate drugs or doses considering the patient’s organ function. The study also defined ‘regular drug’ as a prescribed medication to be taken on schedule, excluding drugs taken only when symptoms occurred.

### 2.6 Data assessment

#### 2.6.1 Data availability and collection

After the survey period, records were collected from the JSPPCS office.

#### 2.6.2 Data analysis

Descriptive analyses were performed to characterize the study population. Bivariate analyses were employed to examine the differences in demographic characteristics using *t*-tests for continuous variables and chi-square tests or Fisher’s exact probability tests for categorical variables. The study also analyzed the relationship between pharmacist interventions, of pharmacists both with or without Board Certified Pharmacist in Palliative Pharmacy (BCPPP), and the rate of improvement in ADEs in patients who used or did not use opioids. All data were analyzed using SPSS software (version 22.0; SPSS Inc., Chicago, IL, United States). Statistical significance was set at *p* < 0.05.

## 3 Results

### 3.1 Characteristics of 50 hospital pharmacists

The background information on pharmacists is presented in Table 1. The median pharmacist’s experience was 14 years. The median number of beds in hospitals where the pharmacists worked was 380, and the average number of beds for which the pharmacists were responsible was 49. During the study period, the pharmacists worked an average of 8.8 h per day, with an average of 4.0 h spent on clinical practice for patients. Fifty-four pharmacists (54.0%) had a BCPPP accredited by the JSPPCS.

**TABLE 1:** Characteristics of the pharmacists included in this study (*n* = 50).

	*n*	(%)
Sex		
Male	27	54.0
Female	23	46.0
Pharmacist experience, years	14 [10.25–19]	
Median [interquartile range]		
Number of beds in charge wards	49 [39–91]	
Median [interquartile range]		
Number of hospital beds	380 [199–652]	
Median [interquartile range]		
Working hours per day	8.8 [1.9]	
Mean [standard deviation]		
Working hours per day spent on medication counseling per day	4.0 [3.0]	
Mean [standard deviation]		
Time spent on continuing education related to the clinical pharmacy in the last year	60 [36–100]	
Median [interquartile range]		
Board pharmacy certification		
No	11	22.0
Yes	39	78.0
BCPPP	27	54.0
BCPOP	11	22.0
JOP	8	16.0
APACC	4	8.0
Others	22	44.0

APACC, accredited pharmacist of ambulatory cancer chemotherapy; BCPPP, board certified pharmacist in palliative pharmacy; BCPOP, board certified pharmacist in oncology pharmacy; JOP, Japanese Society of Pharmaceutical Healthcare and Sciences (JSPHCS) certification of Oncology Pharmacist.

### 3.2 Hospital pharmacist interventions

Hospital pharmacists’ interventions are shown in Table 2. A total of 1,415 patients underwent interventions by 50 pharmacists. More than 80% of the patients had cancer. Out of the total 1,415 patients, 347 individuals (24.5%) received pharmacist interventions specifically aimed at reducing medication usage or decreasing the dosage of their current medications. Of the 347 patients, pharmacists provided 391 deprescribing suggestions. The most common reasons for IMs identified by pharmacists were “long-term administration of irresponsible or aimless medications” (44.5%), “adverse effects caused by medications” (31.5%), and “medications-mediated duplication of the pharmacological effect” (15.3%). Approximately 90% of all pharmacists’ recommendations were accepted for each reason to reduce medications, and consequently, physicians accepted 368 recommendations (94.1%). The average number of regular medications used by patients involved in drug reduction was 8.2 [standard deviation (S.D.): 3.5], and the average number of medications reduced was 1.7 (S.D. 1.4).

**TABLE 2:** Pharmacist contributions in reducing inappropriate medication.

		*n*	(%)
Number of patients involved in the pharmacy service		1415	
Number of pharmacist contributions due to inappropriate prescription			
Patients		347	24.5
Cases		391	
Number of regularly used drugs in patients with inappropriate prescription		8.2 [3.5]	
Mean [standard deviation]			
Reasons and acceptance rates of pharmacist recommendations to reduce medications			
	Recommendations	Acceptance	Acceptance rate
Long-term administration of irresponsible or aimless medications	174	165	94.8
Adverse effects caused by medications	123	116	94.3
Medication-mediated duplication of the pharmacological effect	60	56	93.3
Medication-induced drug–drug interactions	13	12	92.3
Inappropriate drugs for older patients	12	11	91.7
Inappropriate drugs or dose considering the patient’s organ function	9	8	88.9
Total	391	368	94.1
Contents of deprescribing			
Discontinuation		523	
Dose reduction		36	
Average number of discontinued drugs		1.7	
Average number of drugs reduced in doses		0.6	
Average number of drugs discontinued or reduced in dose		1.8	

### 3.3 Characteristics of 347 patients with pharmacy interventions

The backgrounds of the patients who underwent the intervention are presented in Table 3. The most common age group encompassed 310 patients (89.3%) in their 60 s or older. Cancer was the most common disease (297 patients, 85.6%), followed by cardiovascular (15 patients, 4.3%) and urological (7 patients, 2.0%) diseases. Twelve patients (42.7%) received at least one treatment during their current hospitalization.

**TABLE 3:** Characteristics of the patients included in this study (*n* = 347).

		*n*	(%)
Age	90 s	17	4.9
	80 s	109	31.4
	70 s	117	33.7
	60 s	67	19.3
	50 s	25	7.2
	40 s	9	2.6
	30 s	3	0.9
Disease^a^	Cancer	297	85.6
	Cardiovascular disease	15	4.3
	Urological disease	7	2.0
	Chronic kidney disease	5	1.4
	Cerebral nerve disease	5	1.4
	Digestive system disease	3	0.9
	Asthma	3	0.9
	Diabetes	2	0.6
	Hypertension	1	0.3
	Other	21	6.1

^a^ Multiple diseases were recorded in one patient.

### 3.4 Drugs discontinued following pharmacist recommendations

A total of 523 drugs were discontinued following the recommendations of 50 pharmacists during the study period, as shown in Table 4. The most common drug categories, according to the Anatomical Therapeutic Chemical Classification, were the alimentary tract and metabolism (32.3%), nervous system (22.8%), and cardiovascular system (11.3%). The most common drug categories according to functional classification were gastrointestinal medications (14.0%), analgesics (13.2%), and antipsychotics (8.2%). Table 4 also shows the pharmacological categories of drugs that were discontinued following pharmacists’ recommendations.

**TABLE 4:** Medications discontinued following pharmacist recommendations (*n* = 523).

	*n*	(%)
Anatomical therapeutic chemical classification		
Alimentary tract and metabolism	169	32.3
Nervous system	119	22.8
Cardiovascular system	59	11.3
Musculoskeletal system	40	7.6
Blood and blood forming organs	15	2.9
Anti-infectives for systemic use	14	2.7
Respiratory system	13	2.5
Genito urinary system and sex hormones	10	1.9
Systemic hormonal preparations, excluding sex hormones and insulins	8	1.5
Dermatological	6	1.1
Antineoplastic and immunomodulating agents	4	0.8
Various	66	12.6
Functional classification of drug discontinued		
Gastrointestinal medications	73	14.0
Analgesics	69	13.2
Antipsychotics	43	8.2
Hypnotic sedatives	42	8.0
Antiemetics	33	6.3
Laxatives	24	4.6
Others	239	45.7
Discontinued drug according to pharmacological category		
Gastrointestinal medications	73	14.0
Proton pump inhibitors	19	3.6
Prostaglandin analogs	14	2.7
Antiflatulents	11	2.1
Histamine H_2_ receptor blockers	10	1.9
Dopamine receptor antagonists	4	0.8
Mucosal protection agents	1	0.2
Other	16	3.1
Analgesics	69	13.2
Non-steroidal anti-inflammatory drugs	25	4.8
Opioids	20	3.8
Tramadol	11	2.1
Acetaminophen	9	1.7
Analgesic adjuvants	4	0.8
Antipsychotics	43	8.2
Typical antipsychotics	13	2.5
Atypical antipsychotics	11	2.1
Selective serotonin reuptake inhibitors	8	1.5
Other antidepressant	4	0.8
Tricyclic antidepressants	3	0.6
Dementia drugs	1	0.2
Antiepileptic drugs	1	0.2
Antihistamines	1	0.2
Other	1	0.2
Hypnotic sedatives	42	8.0
Benzodiazepines	27	5.2
Non-benzodiazepines	9	1.7
Other	6	1.1
Antiemetics	33	6.3
Dopamine receptor antagonists	31	5.9
Antihistaminic agents	1	0.2
5HT_3_ inhibitors	1	0.2
Laxatives	24	4.6
Salt-based laxative	12	2.3
Peroral stimulative laxatives	5	1.0
μ-receptor antagonist	3	0.6
Chloride channel agonists	2	0.4
Sugar laxatives	1	0.2
Other	1	0.2
Others	239	45.7
Antihypertensive drugs	20	3.8
Vitamins	20	3.8
Diuretics	19	3.6
Herbal medicines	17	3.3
Anticoagulants	15	2.9
Antibacterials	14	2.7
Diabetic drugs	13	2.5
Anti-diuretic drugs	10	1.9
Airway mucus regulators	10	1.9
Hyperlipidemic drugs	9	1.7
Steroids	8	1.5
Ca replacement drugs	6	1.1
Iron replacement drugs	6	1.1
Beta-blockers	4	0.8
Antiallergic drugs	4	0.8
Anti-cancer drugs	4	0.8
Anti-arrhythmic drugs	4	0.8
Hepatobiliary protection agents	4	0.8
Enteral feeding agents	3	0.6
Antidiarrheals	3	0.6
Antitussives	3	0.6
Rehydration agents	3	0.6
Circulation improvers	3	0.6
K-replacement medications	2	0.4
Muscle relaxants	2	0.4
Topical steroids	2	0.4
Other	29	5.5

### 3.5 Improved ADEs due to pharmacy interventions

Table 5 shows the ADEs potentially avoided according to pharmacists’ recommendations. Out of the 123 pharmacy interventions for IMs that considered potential ADEs, physicians accepted 116 of these interventions (94.3%). The three most common ADEs were “hypnotic sedation” (12.2%), “delirium” (8.9%), and “hypotension” (8.9%). Of the 123 symptoms, 55 (44.7%) improved after deprescription. Improve rates of the top three symptoms of ADEs reduced due to pharmacist interventions were “hypnotic sedation” (60.0%), “delirium” (63.6%), and “hypotension” (45.5%).

**TABLE 5:** Adverse drug events potentially avoided due to pharmacist’s recommendations.

		*n*	(%)	Improve	Improving rate (%)
Overall		123		55	44.7
Hypnotic sedation		15	12.2	9	60.0
Delirium		11	100.0	7	63.6
Hypotension		11	73.3	5	45.5
Hemorrhage		8	72.7	1	12.5
Diarrhea		7	87.5	5	71.4
High K blood bed		7	100.0	1	14.3
Extrapyramidal disorders		6	85.7	3	50.0
Nausea		5	83.3	5	100.0
Contraindications		5	100.0	0	0.0
Renal impairment		5	100.0	1	20.0
Low blood sugar		5	100.0	0	0.0
Hypermagnesemia		4	80.0	2	50.0
Gastrointestinal symptoms		4	100.0	1	25.0
Constipation		4	100.0	3	75.0
Liver damage		3	75.0	1	33.3
Respiratory depression		2	66.7	1	50.0
Restlessness		2	100.0	1	50.0
Insomnia		2	100.0	1	50.0
QT prolongation		1	50.0	0	0.0
Malignant syndrome		1	100.0	1	100.0
Pseudo hyperaldosteronism		1	100.0	0	0.0
Thrombocytopenia		1	100.0	0	0.0
Hypercalcemia		1	100.0	0	0.0
Hyperglycemia		1	100.0	0	0.0
Aspiration		1	100.0	0	0.0
Dehydration		1	100.0	0	0.0
Electrolyte abnormalities		1	100.0	0	0.0
Serotonin syndrome		1	100.0	1	100.0
Gastrointestinal symptoms		1	100.0	1	100.0
Dehydration		1	100.0	1	100.0
Hypokalemia		1	100.0	0	0.0
Falling over		1	100.0	1	100.0
Benzodiazepine dependence		1	100.0	1	100.0
Myoclonus		1	100.0	1	100.0
Vertigo		1	100.0	1	100.0

### 3.6 Differences between cancer patients using and not using opioids

Table 6 presents a comparison between the 391 interventions performed by pharmacists on patients who used opioids with those who did not. All patients receiving opioid analgesics suffered from cancer. The average number of regularly used medications was significantly higher among opioid-using patients than among non-opioid-using patients (8.9 vs. 7.3, *p* < 0.001). The average number of deprescribed medications was significantly higher among opioid-using patients than among opioid-non-using patients (1.9 vs. 1.5, *p* < 0.01). There were 210 pharmacy interventions in 180 opioid-using cases and 181 pharmacy interventions in 167 non-opioid-using cases. The top three reasons for IMs were “long-term administration of irresponsible or aimless medications” (*n* = 99), “adverse effects caused by medications” (*n* = 65), and “medication-mediated duplication of the pharmacological effect” (*n* = 33) in opioid-using patients. In patients who did not use opioids, the top three reasons for IMs were similar to those for pharmacy interventions in opioid-using patients. The acceptance rate of opioid users was significantly higher than that of non-opioid users (96.7% vs. 91.2%, *p* = 0.02). The rate of symptom improvement after deprescription was also significantly higher in opioid-using patients than in non-using patients (62.5% vs. 25.0%, *p* < 0.001).

**TABLE 6:** Comparison of interventions implemented by pharmacists in patients using opioids and those not using opioids.

Number of patients who had pharmacy recommendations	Opioid using			Non-opioid using			*p*-value
	*n* = 180			*n* = 167			
Average number of medicines (S.D.)	8.9 (3.7)			7.3 (3.4)			<0.001
Average number of reduced medicines (S.D.)	1.9 (1.4)			1.5 (1.2)			<0.01

Pharmacy recommendations	*n*	Accepted	(%)	*n*	Accepted	(%)	*p*-value
Overall	210	203	96.7	181	165	91.2	0.02
Long-term administration of irresponsible or aimless medications	99	93	93.9	75	69	92.0	0.84
Adverse effects caused by medications	65	54	83.1	58	52	89.7	0.42
Medication-mediated duplication of the pharmacological effect	33	31	93.9	27	25	92.6	0.75
Medication-induced drug–drug interactions	5	4	80.0	8	8	100.0	0.76
Inappropriate drugs for older patients	5	5	100.0	7	6	85.7	0.58
Inappropriate drugs or dose considering the patient’s organ function	3	3	100.0	6	5	83.3	0.66

Rate of symptom improvement due to pharmacy recommendations	*n*	Symptom improvement	(%)	*n*	Symptom improvement	(%)	*p*-value
Number of prescriptions changed by recommendations	64	40	62.5	52	13	25.0	<0.001

S.D., standard deviation.

### 3.7 Differences between BCPPP and non-BCPPP

Table 7 presents a comparison of 391 interventions conducted by pharmacists with and without the BCPPP. The average number of regularly used and reduced medicines due to pharmacy interventions was not significantly different between patients who received pharmacy interventions by the BCPPP and those who received pharmacy interventions by the non-BCPPP. The top three reasons for the IMs detected by BCPPP were “long-term administration of irresponsible or aimless medications” (*n* = 104), “adverse effects caused by medications” (*n* = 83), and “medication-mediated duplication of the pharmacological effect” (*n* = 41). In the non-BCPPP pharmacy interventions, the top three reasons for IMs were similar to those of the BCPPP pharmacy interventions. In addition, the acceptance rate by doctors was not significantly different between pharmacy interventions using the BCPPP and those using the pharmacy interventions by non-BCPPP (94.8% vs. 92.8%, *p* = 0.41). The rate of improvement in symptoms after deprescribing was also significantly higher in pharmacy interventions by the BCPPP than in pharmacy interventions by the non-BCPPP (52.5% vs. 30.6%, *p* = 0.04).

**TABLE 7:** Comparison interventions conducted by pharmacists with and without BCPPP.

Number of patients who had pharmacy recommendations	BCPPP (*n* = 27)			Non-BCPPP (*n* = 23)			*p*-value
	*n* = 225			*n* = 122			
Average number of medicines (S.D.)	8.0 (3.5)			8.5 (3.8)			0.20
Average number of reduced medicines (S.D.)	1.7 (1.3)			1.8 (1.4)			0.82

Pharmacy recommendations	*n*	Accepted	(%)	*n*	Accepted	(%)	*p*-value
Overall	252	239	94.8	139	129	92.8	0.41
Long-term administration of irresponsible or aimless medications	104	99	95.2	70	66	94.3	0.93
Adverse effects caused by medications	83	80	96.4	40	36	90.0	0.30
Medication-mediated duplication of the pharmacological effect	41	38	92.7	19	18	94.7	0.79
Medication-induced drug–drug interactions	11	10	90.9	2	2	100.0	0.84
Inappropriate drugs for older patients	9	8	88.9	3	3	100.0	0.75
Inappropriate drugs or dose considering the patient’s organ function	4	4	100.0	5	4	80.0	0.55

Rate of symptom improvement due to pharmacy recommendations	*n*	Symptom improvement	(%)	*n*	Symptom improvement	(%)	*p*-value
Number of prescriptions changed by recommendations	80	42	52.5	36	11	30.6	0.04

BCPPP, board certified pharmacist in palliative pharmacy; S.D., standard deviation.

## 4 Discussion

This was the first multicenter prospective observational study conducted in Japan to demonstrate how hospital pharmacists contribute to eliminating polypharmacy among inpatients. Of the 1,415 patients involved in the study, the pharmacists recommended discontinuing or reducing the dosage for 347 patients (24.5%). A total of 391 patients received recommendations from pharmacists’ to reduce IMs use, of which 368 (94.1%) were accepted. A total of 523 drugs were discontinued following the pharmacist’s recommendations. Pharmacist intervention improved hypnotic sedation, delirium, and hypotension. Although the trends in IMs were similar to those of a previous questionnaire survey (Uchida et al., 2019), we were able to reveal the results of medication reduction that the questionnaire survey could not clarify. The average number of 8.0 regular medications may reflect the actual situation of patients with cancer in clinical practice. This also reveals polypharmacy and the fact that pharmacists can reduce the number of medications. The pattern of deprescribing due to pharmacy intervention in the questionnaire survey (Uchida et al., 2019) differed from that in the current prospective observational study, indicating a difference between responses based on general knowledge and cases encountered in actual clinical practice. The main reasons for drug reduction were “less meaningful drug use” and “ADEs,” which were apparently associated with patient-related disadvantages. This result also differs from that of a questionnaire survey (Uchida et al., 2019). However, our prospective study of community pharmacists confirmed a similar trend (Uchida et al., 2022). Compared with prospective studies of interventions by community pharmacists, the rate of prescribing interventions by hospital pharmacists was clearly higher and more diverse. This observation could be attributed to the closer working relationships between hospital pharmacists and prescribing doctors. The proximity and collaboration between hospital pharmacists and physicians may have facilitated prescribing interventions, making it easier for hospital pharmacists to intervene compared to pharmacists working in community pharmacies.

This study provided a case report form through which hospital pharmacists could document whether the symptoms of ADEs improved after the intervention. Although the symptoms of ADEs varied widely, approximately 40% of the patients’ adverse symptoms improved after the intervention. Interventions aimed at reducing ADEs are the mainstay of IMs use. A multicenter study in Japan reported that 29 of 100 hospitalized patients had ADEs, of which 4.9% were severe and 1.6% were life-threatening (Morimoto et al., 2011). Patients who experience drug reactions have higher mortality rates and longer hospital stays than those who do not (Bond and Raehl, 2006). Half of the adverse drug reactions are preventable (Chan et al., 2001; Leendertse et al., 2008; Zed et al., 2008), and this intervention can improve patient quality of life, healthcare costs, and treatment. Especially in older individuals, renal dysfunction is a cause of adverse drug reactions due to unintentional overdose caused by delayed elimination of many drugs, such as water-soluble antibacterials, diuretics, and non-steroidal anti-inflammatory drugs (Mangoni and Jackson, 2004). A prospective observational study reported that the most common types of drugs causing adverse drug reactions were anti-infectives, steroids, anticoagulants, non-steroidal anti-inflammatory drugs, and diuretics (Geer et al., 2016). In this study, the reasons for drug reduction varied, leading to a corresponding reduction in the number of medications prescribed to patients. The diversity of reasons contributed to the decision to decrease the usage or dosage of specific drugs in order to address various concerns or optimize the patients’ medication regimens. ADEs were also diverse; however, because many of the patients were in the palliative medicine field, such as oncology, symptom improvement in hypnotic sedation, hypotension, and delirium were achieved in many cases.

Although the number of drug-drug interactions in the breakdown of IMs was small, the acceptance rate was high. Drug interactions are a potential concern for most medications, but it is important to note that the majority of interactions are not absolute contraindications. Instead, they often serve as guidelines for cautious administration. The use of medications with cautious administration can indeed present challenges, and even with knowledge and expertise, it can be difficult for pharmacists to determine in actual practice whether a patient’s condition is adversely affected by a drug interaction or whether to continue a medication that has been identified as causing a potential drug interaction. Although the number of interactions identified by pharmacists in this study was low, the acceptance rate by doctors was high because the interactions identified by pharmacists were clearly unfavorable to the patient. A Swedish drug registry study reported a strong relationship between the number of drugs and drug-drug interactions (Hajjar et al., 2007; Johnell and Klarin, 2007). Since the average number of regularly used drugs in the study was 8.2, it is expected that the number of drug-sensory interactions was high and consequently the associated potential for patient disadvantage was also high. In polypharmacy, drug interactions are more potentially concerning, and education or training on how to deal with them is necessary. Therefore, in Japan, continuing education to detect drug-drug interactions in routine clinical practice is necessary. As suggested by Scott et al., incorporating education on polypharmacy and deprescribing into pharmacists’ training would be advantageous for the success of future pharmacists (Scott et al., 2023).

Pharmacists reduced the number of drugs used in various pharmacological categories. The most common drugs discontinued following pharmacists’ recommendations were gastrointestinal medications (14.0%), followed by analgesics (13.2%), antipsychotics (8.2%), and hypnotic sedatives (8.0%). This was due to long-term administration of irresponsible or aimless medications, adverse drug reactions, duplication of the same type of drug, and indiscriminate prescription. Sleepiness or sedation, cognitive function, and other symptoms improved after discontinuation of the medications. These interventions resulted in the resolution of the ADEs. Our data suggest that the clinical services provided by pharmacists can contribute to reducing the disadvantages faced by outpatients.

Although most patients targeted for pharmacist intervention in this study were cancer patients, it was clear that those using opioids had a higher number of medications and were more likely to be able to reduce their medications. Since the JSPPCS conducted the study, half of the pharmacists who participated in the study had BCPPP, and most of the interventions were for patients with cancer. Comparing patients with and without opioid use showed that patients prescribed opioids use more medications, contributed more to medication reduction, and had a greater rate of improvement in adverse event symptoms. Although pharmacists must consider the use of opioids, the results clearly demonstrate their significance. When comparing pharmacists’ interventions with and without the BCPPP, there was no statistical difference in the number of drugs used between the two groups and no difference in the mean value of medication reduction. There was no statistical difference in the acceptance rate of interventions for IM, but there was a twofold difference in the number of suggestions made by the 27 pharmacists with BCPPP and the 23 without, 252 and 139, respectively. Furthermore, the rate of ADEs improvement was considerably higher in the BCPPP group than in the non-BCPPP group. Hence, the training and certification in BCPPP provided by the JSPPCS holds remarkable value. However, since these results were based on a univariate sub-analysis, a carefully designed prospective intervention study with or without BCPPP is needed to confirm this important clinical question.

Several definitions of inappropriate prescribing exist (O’Connor et al., 2012; O’Mahony et al., 2015; Panel et al., 2015; Levy, 2017; Chun et al., 2018), including the American Geriatrics Society Beers Criteria (Panel et al., 2015) and the Screening Tool of Older People’s Prescriptions (STOPP) (O’Mahony et al., 2015) which are well-known criteria that address multiple elements to reduce polypharmacy. However, to alleviate the burden on hospital pharmacists in their daily practice, we did not use international criteria to detect IMs in this prospective study. Hamilton et al. reported that potential inappropriate medications, as defined by the STOPP criteria, were extensively associated with avoidable drug reactions in older patients (Hamilton et al., 2011). Although the Beers criteria (Panel et al., 2015), the STOPP/START criteria (O’Mahony et al., 2015), and the Medication Appropriateness Index (Hanlon and Schmader, 2013) have clear evidence and should be used for evaluation, only a few facilities in Japan currently implement these criteria in their daily clinical practice. In addition, the use of these criteria requires diagnostic studies and imaging evaluations that pharmacists cannot perform regularly. Therefore, we did not use these criteria in this study. This is further because a Dutch study on outpatients reported that most drug-related problems were not associated with the STOPP/START criteria (Verdoorn et al., 2015); those criteria are not absolute. These criteria should be carefully selected according to the purpose of the study. As the purpose of our study was to clarify pharmacists’ drug reduction activities in actual clinical practice, the study was successful without evaluation using international standard criteria. Polypharmacy is often defined in numbers (Levy, 2017; Masnoon et al., 2017; Wastesson et al., 2018), but there is no unanimous definition of what constitutes polypharmacy (Masnoon et al., 2017). In our study, we define polypharmacy as the use of more drugs than clinically indicated rather than by a specific number (Fulton and Allen, 2005). ADEs cause some symptoms related to the central nervous system and the gastrointestinal tract, often resulting in the prescription of additional drugs to control these symptoms (Budnitz et al., 2011; Carroll and Hassanin, 2017; Wastesson et al., 2018), which is a necessary treatment for patients. Therefore, pharmacists must propose the use of necessary drugs based on the successive assessment of the patient’s treatment and not just the number of drugs. Prescribing multiple medications can negatively impact patient adherence and health-related quality of life (Schenker et al., 2019); it has been reported that medication adherence decreases with the number of medications prescribed (Pasina et al., 2014), making the involvement of pharmacists’ rather crucial.

This prospective study proved that pharmacists could contribute to improving the use of IMs in clinical practice in Japan. This study, however, has few limitations, including the possibility of bias in that most registered pharmacists were JSPPCS members, the small number of participating pharmacists, and the level of interest in polypharmacy among the participating pharmacists. Nonetheless, the results obtained in this study were not influenced by these limitations as they provide a background similar to the current situation in Japan, where there are many older patients suffering from cancer. While it is important to strive for minimizing bias in study design, it is worth noting that complete elimination of bias may not be feasible in certain prospective studies that involve real-world clinical data. We designed this study to minimize the burden of clinical trials by considering the work of pharmacists. In the data analysis, since the pharmacist intervention in actual clinical practice was summarized, its detailed analysis was not designed before the study, and the data obtained could not be analyzed in detail due to the study design, which is similar to a fact-finding survey. Therefore, subsequent studies are required to collect more detailed data. Alternatively, it is desirable to use a study design that clearly demonstrates the effectiveness of pharmacist intervention, such as a two-arm comparison with and without pharmacist intervention, or a pre- and post-pharmacist intervention comparison, rather than a single-arm observational study design. However, pharmacist intervention is a standard practice within Japanese medical care, and it is considered unethical for medical institutions to intentionally create an intervention group without including pharmacist intervention. Unfortunately, the more we aim to collect detailed data, the greater becomes the burden, drifting us away from actual clinical results. In addition, as there are reports of improvement in IMs by multidisciplinary teams led by nurses overseas (Garland et al., 2021), such clinical work should be conducted together with physicians, nurses, and other multidisciplinary teams (Liu, 2014); in terms of collaborative work, the role of clinical pharmacists in Japan is still in the process of development and may not be considered mature. In addition, because of the importance of working with patients to resolve polypharmacy and IMs, Woodward suggested deprescribing principles by reviewing all current medications, identifying medications for discontinuation, planning a deprescribing regimen, and working with patients and caregivers (Woodward, 2003). Therefore, a comprehensive approach is required.

## 5 Conclusion

This multicenter prospective observational study conducted in Japan is the first to demonstrate the effectiveness of hospital pharmacist interventions in promoting appropriate prescriptions. As a result, there was a significant reduction in the number of medications used and a decrease in polypharmacy. The study also highlighted the active role of hospital pharmacists in addressing polypharmacy by discontinuing inappropriate drugs or reducing the dosage of regularly prescribed medications, which contributed to the mitigation of ADEs.

## Data Availability

The datasets presented in this article are not readily available because this dataset only to raw, anonymized data. Never share participants identifiable data. Requests to access the datasets should be directed to ssuzuki@east.ncc.go.jp.
